# Intestinal Morphologic and Microbiota Responses to Dietary *Bacillus* spp. in a Broiler Chicken Model

**DOI:** 10.3389/fphys.2018.01968

**Published:** 2019-01-17

**Authors:** Cheng-liang Li, Jing Wang, Hai-jun Zhang, Shu-geng Wu, Qian-ru Hui, Cheng-bo Yang, Re-jun Fang, Guang-hai Qi

**Affiliations:** ^1^College of Animal Science and Technology, Hunan Agricultural University, Changsha, China; ^2^Key Laboratory of Feed Biotechnology of Ministry of Agriculture and Rural Affairs, Feed Research Institute, Chinese Academy of Agricultural Sciences, Beijing, China; ^3^Department of Animal Science, Faculty of Agricultural and Food Sciences, University of Manitoba, Winnipeg, MB, Canada

**Keywords:** probiotics, growth performance, intestinal morphology, jejunum microbiota, broiler

## Abstract

Dietary inclusion of probiotic *Bacillus* spp. beneficially affect the broiler chickens by balancing the properties of the indigenous microbiota causing better growth performance. The effects of three *Bacillus* spp. on the growth performance, intestinal morphology and the compositions of jejunal microflora were investigated in broiler chickens. A total of 480 1-day-old male Arbor Acres broilers were randomly divided into four groups. All groups had six replicates and 20 birds were included in each replicate. The control birds were fed with a corn-soybean basal diet, while three treatment diets were supplemented with *Bacillus coagulans* TBC169, *B. subtilis* PB6, and *B. subtilis* DSM32315 with a dosage of 1 × 10^9^ cfu/kg, respectively. The experiment lasted for 42 days. The compositions and diversity of jejunal microflora were analyzed by MiSeq high-throughput sequencing. The *B. coagulans* TBC169 group showed marked improvements of growth performance, nutrient digestibility and intestinal morphology compared with the other *B. subtilis* treatments. *B. coagulans* TBC169 supplementation improved the average body weight (BW), average daily weight gain (ADG), total tract apparent digestibility of crude protein and gross energy (GE), and reduced feed conversion rate (FCR) compared with the control group (*P* < 0.05). The villus height to crypt depth ratio (VH/CD) of jejunum and duodenum was increased in the birds fed with *B. coagulans* TBC169 compared with the control group (*P* < 0.05). However, two *B. subtilis* treatments presented more positive variation of the jejunum microflora of chickens than that in the *B. coagulans* TBC169 group. *B. subtilis* PB6 and *B. subtilis* DSM32315 treatments improved the diversity of jejunal microbiota on day 21 compared with the control (*P* < 0.05), while which were decreased on day 42 (*P* < 0.05). The supplementation with *B. coagulans* TBC169 significantly improved the proportion of Firmicutes, otherwise two *B. subtilis* significantly improved the proportion of Proteobacteria, Bacteroidetes, Actinobacteria, and Acidobacteria at the phylum level during starter phase and decreased the proportion of Bacteroidetes during growing phase compared with the control. The supplementation with *B.subtilis* DSM32315 significantly improved the proportion of Clostridiales during starter phase, whereas two *B. subtilis* significantly improved the proportion of *Pseudomonas, Burkholderia, Prevotella, DA101* during growing phase at the genus level compared with the control. In conclusion, the dietary supplementation with probiotic *Bacillus* spp. strains improved body weight and intestinal morphology in broiler chickens, which might be associated with the gut microbiota.

## Introduction

Broiler chickens have been reared and consumed widely around the world since they can provide high-quality meat and eggs for human beings. During the last 6 or 7 decades, with the development of production system, chickens can convert feed into muscle mass efficiently (Clavijo and Flórez, 2017). The worldwide demand for chicken meat continues to grow considerably (Tallentire et al., 2018). Meanwhile, because of the detrimental side effects of antibiotics on both poultry products and human well-being, an increasing number of countries have implemented the withdrawal of antibiotic growth promoters (AGPs), which was previously recognized as growth promoters to boost animal growth performance and inhibit the spread of certain diseases (Mashayekhi et al., 2018).

Probiotics are non-pathogenic bacterial cultures that can adjust intestinal microflora and in turn improve the gastrointestinal environment of the host. In addition, probiotics have positive impacts on colonized beneficial bacterial and growth performance in broilers and pigs (Jeong and Kim, 2014; Valeriano et al., 2017). Noticeably, probiotics, as the alternatives for antibiotics used to prevent poultry diseases and improve production performance, have been demonstrated to be beneficial to chickens’ growth performance and health, such as the increases of body weight (BW), feed conversion efficiency, immune response, resistance to bacterial infection, and regulation of intestinal microflora (Xu et al., 2012; Cao et al., 2013; Song et al., 2014; Clavijo and Flórez, 2017; Haque et al., 2017; Hussain et al., 2017a; Azad et al., 2018; Mashayekhi et al., 2018).

Presently, spore-forming bacteria, such as *Bacillus* spp. including *Bacillus subtilis, B. coagulans*, and *B. licheniformis* etc. have been widely used as commercialized probiotic products for humans and animals (Barbosa et al., 2005; Zhang et al., 2013; Park and Kim, 2014; Haque et al., 2017; Xu et al., 2017). *Bacillus* spp. have been also considered to be promising probiotics, due to the high stability of spores, which is resistant to high temperature and harsh gastrointestinal conditions during feed processing and that can confer health benefits to the host (Mazanko et al., 2017). Previous study showed that *B. subtilis* and *B. coagulans* had positive effects on tilapia growth and immune response (Zhou et al., 2010). Moreover, it has been reported that dietary supplementation with *B. subtilis* exerted a beneficial role in the digestibility and intestinal microbes of weaning piglets, and finally improving their growth performance (Tsukahara et al., 2013). Lee S.H. et al. (2014) suggested that dietary supplementation with *B. subtilis* in pigs exhibited significant effects on gut morphology, microbiota compositions and immune function. Feeding broilers with *B. coagulans* diets can improve the feed conversion ratio (FCR) and beneficially modulate the composition of the microflora, which markedly enhanced the relative abundance of lactobacilli and tended to lower coliform bacteria composition (Hung et al., 2012). As a result, oral administration with *B. subtilis* and *B. coagulans* may have potential to improve the growth state, intestinal function and microflora compositions of broilers. But many studies reported that probiotics had no significant effect on growth in broilers (Arslan et al., 2004; Chitra et al., 2004; Das et al., 2005).

Researches on the classification and identification of intestinal microbes in poultries were conducted progressively because of the conventional molecular ecology techniques such as denaturing gradient gel electrophoresis (DGGE) fingerprints (Li et al., 2017; Yang et al., 2018). However, these techniques can just detect minority dominant population and it is difficult to study the composition, structure and diversity of microflora. In recent years, with the technical development, the high-throughput sequencing has been promoted widely, which realized the parallel comparison among multiple samples on the level of metagenome, and can detect the microbial diversity including rare species more sensitively (Zhou et al., 2011; Micucci et al., 2017; Yin et al., 2017).

However, as three typical strains of *B. subtilis* and *B.* coagulans, *B. coagulans* TBC169, *B. subtilis* PB6 and *B. subtilis* DSM32315, have been rarely studied as probiotics to improve the well-being of broiler chickens. Furthermore, little is known about the effects of *B. subtilis* and *B. coagulans* on gastrointestinal tract (GIT) microflora compositions and intestinal morphology. Therefore, the objective of this study was to evaluate the effects of *B. coagulans* TBC169, *B. subtilis* PB6 and *B. subtilis* DSM32315 supplementation on the growth performance, nutrient utilization and morphological development of the small intestine in broilers. The microflora compositions in the jejunum of broilers were further studied by MiSeq high-throughput sequencing to reveal the relationship among the growth performance, intestinal morphology and microflora in order to promote new evidences for the mechanism of action of these probiotics.

## Materials and Methods

### Probiotics Strains

Three kinds of commercial probiotics strains were *B. coagulans* TBC169, *B. subtilis* PB6 and *B. subtilis* DSM32315. The probiotic product contains at least 2.0 × 10^9^ cfu/g of *Bacillus* spp. and was stored in a sterilized container. The concentration of each *Bacillus* spp. product was 1 × 10^9^ cfu/kg.

### Experimental Design and Dietary Treatments

A total of 480 healthy 1-day-old male Arbor Acres (Isolauri et al., 2001) broilers (Beijing Huadu Broiler Company, Beijing, China) with average body weight of 48 g were randomly allotted into four treatments. There were six replicates (20 birds per replicate) for each treatment. The diets, without any antibiotics and growth promoters, were based on corn–soybean meal and formulated to meet starter (days 1–21) and grower–finisher (days 22–42) growth requirements (Table 1) (Chinese Feeding Standard of Chicken, Ministry of Agriculture of China, 2004; National Research Council, 1994). Dietary treatments consisted of basal diet with *B. coagulans* TBC169; basal diet with *B. subtilis* PB6; (3) basal diet with *B. subtilis* DSM32315 and the basal diet with no probiotic supplementation was set as the control. Treatments were supplemented with 200 mg/kg *Bacillus* spp. All experimental protocols were approved by Animal Care and Use Committee of the Feed Research Institute of the Chinese Academy of Agricultural Sciences. All management of birds in this study was according to the guideline of raising AA broilers (Delezie et al., 2012).

**Table 1:** Composition and nutrient levels of the basal diet (air-dry basis, %).

Items	Starting (days 1–21)	Growing (days 22–42)
**Ingredients**		
Corn	56.37	63.08
Soybean meal	36.56	29.24
Soybean oil	3.00	3.50
CaHPO_4_	1.24	1.61
Limestone	1.61	1.18
NaCl	0.35	0.35
*DL*-Met	0.27	0.30
*L*-Lys⋅HCl	0.19	0.27
*L*-Thr	0.09	0.15
Vitamin premix^1^	0.02	0.02
Mineral premix^2^	0.20	0.20
50% choline chloride	0.10	0.10
Total	100	100
**Calculated nutrient levels**		
AME (MJ/kg)	12.55	12.97
Crude protein, %	21.00	19.00
Calcium, %	1.00	0.90
Available phosphorus, %	0.45	0.40
Lysine, %	1.15	1.05
Methionine, %	0.55	0.48
Methionine + cystine, %	0.92	0.84
Threonine, %	0.82	0.69
Tryptophan, %	0.24	0.22


*^1^The vitamin premix supplied the following per kg of complete feed: vitamin A, 12,500 IU; vitamin D3, 2,500 IU; vitamin K3, 2.65 mg; vitamin B1, 2 mg; vitamin B2, 6 mg; vitamin B12, 0.025 mg; vitamin E, 30 IU; biotin, 0.0325 mg; folic acid, 1.25 mg; pantothenic acid, 12 mg; niacin, 50 mg. ^*2*^The mineral premix supplied the following per kg of complete feed: Cu, 8 mg; Zn, 75 mg; Fe, 80 mg; Mn, 100 mg; Se, 0.15 mg; I, 0.35.*

### Growth Performance

Body weight and feed intake were recorded (days 1–21 and day 22–42). Average daily feed intake (ADFI), average daily weight gain (ADG), and feed conversion ratio (FCR, feed/weight gain, g/g) were calculated.

### Apparent Total Tract Nutrients Digestibility

All droppings (five pens for each treatment) were sampled daily for 5 consecutive days from day 37 in this study. Dry matter (DM), crude protein (CP), crude ash, calcium (Ca) and phosphorus (P) in the diet and excreta samples were analyzed according to the method of the Association of Official Analytical Chemists International, 2007. Gross energy (GE) of these samples was tested by a bomb calorimetry (Gallenkamp Autobomb, London, United Kingdom).

### Histology and Morphometric Analysis of the Intestine

On days 21 and 42, five chicks close to average weight from each treatment were killed and intestinal sections were fixed to measure the intestinal villus height (VH) and crypt depth (CD) (Sun et al., 2005).

### Sampling, DNA Extraction and PCR (Polymerase Chain Reaction) Amplification

Four jejunum samples each treatment were selected from the five chickens slaughtered above as the next study about microbiota on days 21 and 42. The jejunum was ligated by light twine, removed and finally collected in cryogenic vials. All samples were quickly put into liquid nitrogen and stored at -80°C until DNA extraction. The jejunum content of each group was collected and homogenized for further experiments.

Total jejunal bacterial genomic DNA was extracted from content samples by using the Fast DNA SPIN extraction kits (MP Biomedicals, Santa Ana, CA, United States) following the manufacturer’s instructions. Subsequently, The quantity and quality of extracted DNAs were measured using a NanoDrop ND-1000 spectrophotometer (Thermo Fisher Scientific, Waltham, MA, United States) and agarose gel electrophoresis, respectively. The DNA was used as templates to amplify the V4 hyper variable region of 16S rRNA gene by PCR using barcoded fusion primers [forward primer: 520 (5-AYTGGGYDTAAAGNG-3), reverse primer: 802 (5-TACNVGGGTATCTAATCC-3)]. Sample-specific 7-bp barcodes were incorporated into the primers for multiplex sequencing. The PCR components contained 5 μl of Q5 reaction buffer (5×), 5 μl of Q5 High-Fidelity GC buffer (5×), 0.25 μl of Q5 High-Fidelity DNA Polymerase (5 U/μl), 2 μl (2.5 mM) of dNTPs, 1 μl (10 μM) of each Forward and Reverse primer, 2 μl of DNA Template, and 8.75 μl of ddH2O. Thermal cycling consisted of initial denaturation at 98°C for 2 min, followed by 25 cycles consisting of denaturation at 98°C for 15 s, annealing at 55°C for 30 s, and extension at 72°C for 30 s, with a final extension of 5 min at 72°C. PCR amplicons were purified with Agencourt AMPure Beads (Beckman Coulter, Indianapolis, IN, United States) and quantified using the PicoGreen dsDNA Assay Kit (Invitrogen, Carlsbad, CA, United States). The final sequencing library was prepared by mixing the equal amount of purified PCR products, followed by an end reparation with the addition of a poly (A) tail, and the amplicons were connected with each other with the sequencing adapters.

### MiSeq High-Throughput Sequencing and Analysis

Purified PCR products from the 31 samples were mixed with equal concentrations, which were performed using the Illumina MiSeq platform with MiSeq Reagent Kit v3 at Shanghai Personal Biotechnology Co., Ltd. (Shanghai, China). Sequencing libraries were generated and analyzed according to previous studies (Yin et al., 2017, 2018a,b).

The Quantitative Insights Into Microbial Ecology (QIIME, v1.8.0) pipeline was employed to process the sequencing data, as previously described (Caporaso et al., 2010). Briefly, raw sequencing reads with exact matches to the barcodes were assigned to respective samples and identified as valid sequences. The low-quality sequences were filtered through following criteria (Gill et al., 2006; Chen and Jiang, 2014): sequences that had a length of <150 bp, sequences that had average Phred scores of <20, sequences that contained ambiguous bases, and sequences that contained mononucleotide repeats of >8 bp. Paired-end reads were assembled using FLASH (Magoč and Salzberg, 2011). After chimera detection, the remaining high-quality sequences were clustered into operational taxonomic units (OTUs) at 97% sequence identity by UCLUST (Edgar, 2010). A representative sequence was selected from each OTU using default parameters. OTU taxonomic classification was conducted by BLAST searching the representative sequences set against the Greengenes Database (DeSantis et al., 2006) using the best hit (Altschul et al., 1997). An OTU table was further generated to record the abundance of each OTU in each sample and the taxonomy of these OTUs. OTUs containing less than 0.001% of total sequences across all samples were discarded. To minimize the difference of sequencing depth across samples, an averaged, rounded rarefied OTU table was generated by averaging 100 evenly resampled OTU subsets under the 90% of the minimum sequencing depth for further analysis.

To investigate the diversity of the jejunum microbiota, alpha diversity analysis was made by using the OUT table. Diversity indexes (Shannon, Simpson) (Chao and ShenMing, 1992) were calculated. Sequence data analyses were mainly performed using QIIME and R packages (v3.2.0). OTU-level ranked abundance curves were generated to compare the richness and evenness of OTUs among samples. Beta diversity analysis was performed to investigate the structural variation of microbial communities across samples using UniFrac distance metrics (Lozupone and Knight, 2005; Lozupone et al., 2007) and visualized via principal coordinate analysis (PCoA) and non-metric multidimensional scaling (NMDS) (Ramette, 2007). The significance of differentiation of microbiota structure among groups was assessed by PERMANOVA (McArdle and Anderson, 2001) and ANOSIM (Clarke, 1993; Warton et al., 2012) using R package “vegan”. Taxa abundances at the phylum, class, order, family and genus levels were statistically compared among groups by LEfSe was performed to detect differentially abundant taxa across groups using the default parameters (Segata et al., 2011). PLS-DA was also introduced as a supervised model to reveal the microbiota variation among groups, using the “plsda” function in R package “mixOmics” (Chen et al., 2011). Spearman correlation coefficients were calculated for correlation between growth performance (i.e., BW and ADG) and change of microbiota, which aimed to establish suitable microbial composition for better growth performance.

### Statistical Analysis

Data were analyzed by one-way ANOVA and subsequent Duncan’s multiple range test (SPSS 19.0 for Windows; SPSS Inc., Chicago, IL, United States). Results are expressed as means ± SEM. Probability values of less than or equal to 0.05 (*P* ≤ 0.05) were considered significant, whereas a trend for a treatment effect was noted for *P* ≤ 0.10.

## Results

### Growth Performance

The effects of dietary probiotics supplementation on the growth performance of broilers were shown in Table 2. The supplementation with *B. coagulans* TBC169 in feeds increased BW on days 21 and 42 (*P* < 0.01) and ADG (*P* < 0.01) during starter phase (days 1–21) and the whole phase (days 1–42) compared with the control. The supplementation with *B. subtilis* PB6 in diets increased BW on day 21 (*P* < 0.05). Results showed that compared with *subtilis* PB6 group and *B. subtilis* DSM32315 group, the addition of *B. coagulans* TBC169 in broiler’s diets improved (*P* < 0.05) BW on day 42 and ADG during the overall period (days 1–42). Dietary probiotic supplementations tended to increase the ADG (*P* = 0.086) during grower phase (days 22–42). However, ADFI and FCR were no difference (*P* > 0.05) among the groups of *B. coagulans* TBC169, *B. subtilis* PB6 and *B. subtilis* DSM32315.

**Table 2:** Effect of dietary probiotic supplementation on growth performance of broiler chickens^1,2,3^.

Items^4^	Control	*Bacillus coagulans* TBC169	*Bacillus subtilis* PB6	*Bacillus subtilis* DSM32315	*p*-value
Initial BW (g)	48.16 ± 0.12	47.98 ± 0.21	48.09 ± 0.15	48.08 ± 0.12	0.866
BW on day 21 (g)	918.44 ± 17.11^a^	986.72 ± 19.30^c^	963.93 ± 15.14^bc^	947.54 ± 7.53^abc^	0.035
BW on day 42 (g)	2715.49 ± 34.71^a^	2849.49 ± 86.35^c^	2786.96 ± 52.38^ab^	2741.20 ± 42.35^ab^	0.024
**Starter phase (days 1–21)**					
ADG (g)	41.48 ± 0.84^a^	44.70 ± 0.92^c^	43.61 ± 0.72^ac^	42.83 ± 0.36^ac^	0.039
ADFI (g)	56.08 ± 0.83	56.59 ± 0.73	56.97 ± 0.83	55.30 ± 1.02	0.561
FCR (F/G, g/g)	1.36 ± 0.04	1.27 ± 0.02	1.31 ± 0.02	1.29 ± 0.02	0.142
**Grower phase (days 22–42)**					
ADG (g)	85.58 ± 1.45	94.32 ± 4.17	86.81 ± 2.43	85.41 ± 1.70	0.086
ADFI (g)	149.42 ± 3.89	150.93 ± 5.96	154.22 ± 3.74	144.34 ± 3.56	0.254
FCR (F/G, g/g)	1.75 ± 0.03	1.67 ± 0.05	1.78 ± 0.02	1.69 ± 0.06	0.223
**Whole phase (days 1–42)**					
ADG (g)	63.52 ± 0.83^a^	69.51 ± 2.05^c^	65.21 ± 1.25^ab^	64.12 ± 1.01^ab^	0.024
ADFI (g)	108.25 ± 2.89	108.99 ± 4.51	107.46 ± 2.65	105.16 ± 3.67	0.590
FCR (F/G, g/g)	1.70 ± 0.03	1.61 ± 0.03	1.65 ± 0.02	1.64 ± 0.07	0.536


*^a,b,c^Values within a row with no common superscript differ significantly (P < 0.05). ^1^Groups of chickens fed the corresponding diet. ^2^n = 6 replicates per treatment. ^3^Data are the mean of six replicates with 20 birds each. ^4^BW, body weight; ADG, average daily weight gain; ADFI, average daily feed intake; FCR, feed conversion ratio (feed:gain, g:g).*

### Apparent Total Tract Nutrient Digestibility

Dietary supplementation with different *Bacillus* spp. influenced (*P* = 0.045, *P* = 0.011, respectively) the apparent total tract digestibility of GE and CP during the feeding phase (Table 3). Supplementation with *B. coagulans* TBC169 and *B. subtilis* DSM32315 improved (*P* < 0.05) the apparent total tract digestibility of GE and CP compared with the control group. Supplementation with *B. coagulans* TBC169 increased (*P* < 0.05) the apparent total tract digestibility of CP compared with the *B. subtilis* PB6 group. There were no statistical differences (*P* > 0.05) in the apparent total tract digestibility of DM, Ca and P among groups.

**Table 3:** Effect of dietary probiotic supplementation on the apparent total tract nutrients digestibility in broiler chickens (%)^1^.

Item	Control	*Bacillus Coagulans* TBC169	*Bacillus subtilis* PB6	*Bacillus subtilis* DSM32315	*p*-value
Dry matter	72.48 ± 0.09	72.72 ± 0.98	71.23 ± 0.38	73.14 ± 1.84	0.514
Gross energy	77.31 ± 0.40^a^	79.64 ± 0.94^b^	77.84 ± 0.27^ab^	79.87 ± 0.85^b^	0.045
Crude protein	57.83 ± 0.50^a^	64.31 ± 1.89^c^	59.99 ± 0.83^ab^	63.96 ± 1.50^bc^	0.011
Crude ash	16.18 ± 1.62	24.96 ± 1.01	17.56 ± 0.98	22.20 ± 0.73	0.133
Ca	37.07 ± 2.12	44.06 ± 2.47	38.50 ± 2.38	38.50 ± 4.55	0.330
P	45.42 ± 2.39	49.50 ± 2.84	43.64 ± 1.94	43.73 ± 3.64	0.380


*^a, b,c^Means values in the same row with different letters differ significantly (P < 0.05). ^1^n = 6 replicates per treatment.*

### Intestinal Morphology

**FIGURE 1:**
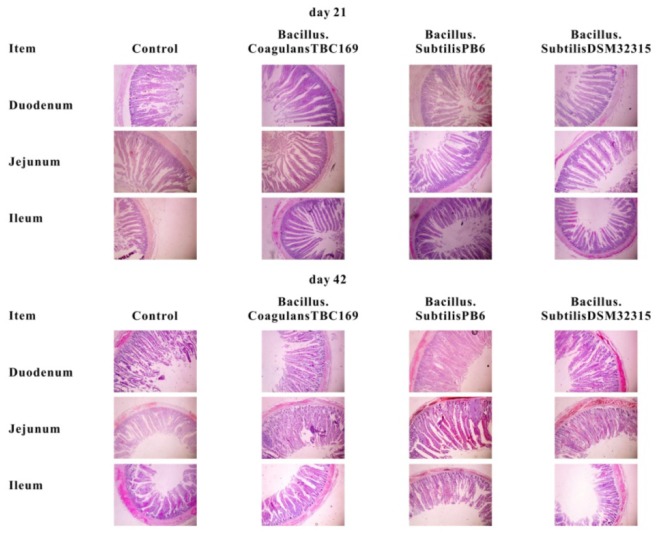
Slice of jejunum morphology (40×) of broiler chickens on day 21 and day 42.

The intestinal morphology of small intestine of broilers in different treatments on days 21 and 42 was shown in Table 4 and Figure 1. On day 21, Dietary supplementation with *Bacillus* spp. tended to influence (*P* = 0.068) the CD of jejunum. The jejunal CD of *B. coagulans* TBC169 group was lower (*P* < 0.05) than those of the control group. Dietary supplementation with *Bacillus* spp. increased (*P* = 0.05) the VH/CD of jejunum. The jejunal VH/CD ratio of *B. coagulans* TBC169 group was higher (*P* < 0.05) than those of the control group. There were no differences (*P* > 0.05) among other groups. On day 42, Dietary supplementation with *Bacillus* spp. influenced (*P* < 0.05) the VH and VH/CD ratio of jejunum. The jejunal VH of *B. coagulans* TBC169 group and *B. subtilis* PB6 group were higher (*P* < 0.05 and *P* < 0.01) than that of the control group. The jejunal VH/CD ratio of *B. coagulans* TBC169 group and *B. subtilis* PB6 group were greater (*P* < 0.05) than that of control group. The jejunal VH of *B. subtilis* PB6 group were higher (*P* < 0.05) than those of *B. coagulans* TBC169 group and *B. subtilis* DSM32315 group. Meanwhile, there were no differences (*P* > 0.05) among other groups. The dietary probiotics supplementation did not affect (*P* > 0.05) the intestinal parameters in the ileum of broilers.

**Table 4:** Effect of dietary *Bacillus* spp. supplementation on the intestinal morphology of broiler chickens on the age of days 21 and 42^1,2^.

	Item	Control	*Bacillus coagulans* TBC169	*Bacillus subtilis* PB6	*Bacillus subtilis* DSM32315	*p-*value
**Day 21**					
Duodenum	Villous	1468.87 ± 111.43	1740.37 ± 55.37	1642.75 ± 121.67	1507.82 ± 79.83	0.259
	Crypt	291.19 ± 36.13	208.18 ± 41.09	223.7 ± 15.5	205.07 ± 34.61	0.236
	Villous/crypt	5.33 ± 0.72	9.3 ± 1.66	7.56 ± 0.88	7.98 ± 1.1	0.122
Jejunum	Villous	1154.92 ± 118.33	1491.31 ± 99.2	1260.29 ± 104.58	1313.77 ± 78.41	0.167
	Crypt	240.49 ± 31.76	145.83 ± 8.16	196.18 ± 24.65	162.34 ± 33.74	0.068
	Villous/crypt	5.23 ± 0.96^a^	10.47 ± 1.16^b^	6.69 ± 0.85^ab^	9.31 ± 2.97^ab^	0.050
Ileum	Villous	883.62 ± 80.68	838.41 ± 106.92	811.3 ± 67.72	774.95 ± 94.7	0.849
	Crypt	178.7 ± 5.47	151.53 ± 13.94	154.58 ± 21.12	133.04 ± 8.52	0.324
	Villous/crypt	4.95 ± 0.46	5.5 ± 0.3	5.41 ± 0.52	5.96 ± 1.1	0.772
**Day 42**					
Duodenum	Villous	1482.97 ± 29.07	1627.7 ± 139.96	1394.45 ± 81.06	1465.38 ± 78.23	0.413
	Crypt	287.63 ± 90.62	227.1 ± 33.3	265.24 ± 16.79	243.82 ± 31.1	0.760
	Villous/crypt	6.17 ± 1.65	7.72 ± 1.14	5.35 ± 0.51	6.27 ± 0.73	0.363
Jejunum	Villous	1019.34 ± 112.86^a^	1354.03 ± 101.56^b^	1493.5 ± 105.49^c^	1080.81 ± 85.51^ab^	0.032
	Crypt	214.93 ± 18.01	197.79 ± 16.48	217.3 ± 34.79	180.93 ± 7.47	0.617
	Villous/crypt	4.74 ± 0.35^a^	6.9 ± 0.41^b^	7.21 ± 1.11^b^	5.97 ± 0.33^ab^	0.042
Ileum	Villous	856.21 ± 66.26	926.34 ± 65.43	867.31 ± 79.72	807.74 ± 68.47	0.761
	Crypt	171.24 ± 21.34	144.34 ± 32.39	187.35 ± 26.6	166.65 ± 26.5	0.766
	Villous/crypt	5.24 ± 0.77	6.98 ± 1.26	4.96 ± 0.77	5.34 ± 0.88	0.503


*^a,b,c^Means with a row with no common superscripts differ significantly (P < 0.05). ^1^Groups of chickens fed the corresponding diet. ^2^n = 5 replicates per treatment.*

### Impact of *Bacillus* spp. Supplementation on Abundance of Microbial Taxa

Based on the V4 region of the 16S rDNA sequence, 114506 amplicons were used for this study with the average of 36936 amplicons for each sample (ranging from 30256 to 47874).

**FIGURE 2:**
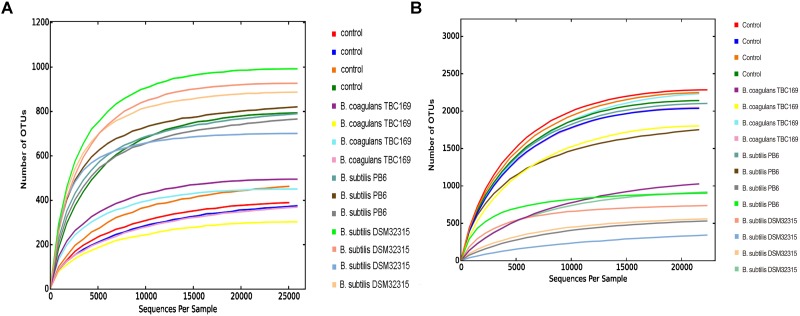
Rarefaction curve of the seven samples. Horizontal axis: the amount of effective sequencing data; vertical axis: the observed number of operational taxonomic units. **(A)** Day 21 and **(B)** day 42.

As shown in Table 5, probiotics increased (*P* < 0.004) the number of OTUs in the jejunum microbiota at five different taxonomic levels (phylum, class, order, family, genus) on days 21 and 42. On day 21, the microbial abundance in broiler chickens in two *B. subtilis* (*B. subtilis* PB6 and *B. subtilis* DSM32315) groups was higher (*P* < 0.01) than *B. coagulans* TBC169 group and the control group, whereas there was no difference (*P* > 0.05) between the *B. coagulans* TBC169 group or the control group at five different taxonomic levels (phylum, class, order, family, genus). Two *B. subtilis strains* (*B. subtilis* PB6 and *B. subtilis* DSM32315) improved (*P* < 0.001) the microbial abundance of jejunum in broiler chickens compared with the *B. coagulans* TBC169 treatment. On day 42, three *Bacillus* spp. (*B. coagulans* TBC169, *B. subtilis* PB6, and *B. subtilis* DSM32315) influenced (*P* < 0.004) the microbial abundance of jejunum in broiler chickens than the control group at different taxonomic levels. The microbial abundance of broiler chickens in *B. subtilis* DSM32315 group was lower (*P* < 0.01) than *B. coagulans* TBC169 group and the control group, whereas there was no difference (*P* > 0.05) between two *B. subtilis* strains. The curves of OTU rank and rarefaction were calculated. The rarefaction curves showed that the total richness of the microbial community of all samples achieved a high sampling coverage (Figure 2).

**Table 5:** The effect of different *Bacillus* spp. on OTUs of gut microbiota of broiler chickens on days 21 and 42^1^.

Items	Control	*Bacillus coagulans* TBC169	*Bacillus.Subtilis* PB6^2^	*Bacillus subtilis* DSM32315	*p*-value
**Day 21**					
Phylum	456 ± 103^a^	383 ± 44^a^	777 ± 15^c^	871 ± 62^c^	0.001
Class	456 ± 102^a^	384 ± 44^a^	773 ± 16^c^	866 ± 62^c^	0.001
Order	428 ± 80^a^	374 ± 40^a^	738 ± 16^c^	816 ± 62^c^	0.000
Family	334 ± 41^a^	324 ± 27^a^	557 ± 20^c^	604 ± 47^c^	0.000
Genus	203 ± 13^a^	212 ± 10^a^	305 ± 7^c^	317 ± 24^c^	0.000
Species	51 ± 6	57 ± 5	52 ± 2	55 ± 4	0.804
Unclassified	50 ± 7^a^	21 ± 5^c^	15 ± 3^cd^	6 ± 1^d^	0.000
**Day 42**					
Phylum	2171 ± 55^a^	1771 ± 263^ab^	1321 ± 365^bcd^	636 ± 123^d^	0.004
Class	2138 ± 54^a^	1748 ± 259^ab^	1308 ± 359^bcd^	633 ± 122^d^	0.004
Order	1911 ± 41^a^	1580 ± 229^ab^	1202 ± 319^bcd^	586 ± 107^d^	0.004
Family	1285 ± 19^a^	1078 ± 145^ab^	788 ± 180^bcd^	439 ± 69^d^	0.002
Genus	582 ± 7^a^	506 ± 59^ab^	387 ± 76^bcd^	242 ± 25^d^	0.002
Species	86 ± 2^a^	77 ± 10^a^	65 ± 12^ac^	39 ± 5^c^	0.010
Unclassified	7 ± 0.3^a^	8 ± 0.4^a^	4 ± 1^c^	4 ± 0.5^c^	0.001


*^a,b,c,d^Means within a row with no common superscripts differ significantly (P < 0.05). ^1^n = 4 replicates per treatment. ^2^n = 3 replicates per treatment on day 21.*

**FIGURE 3:**
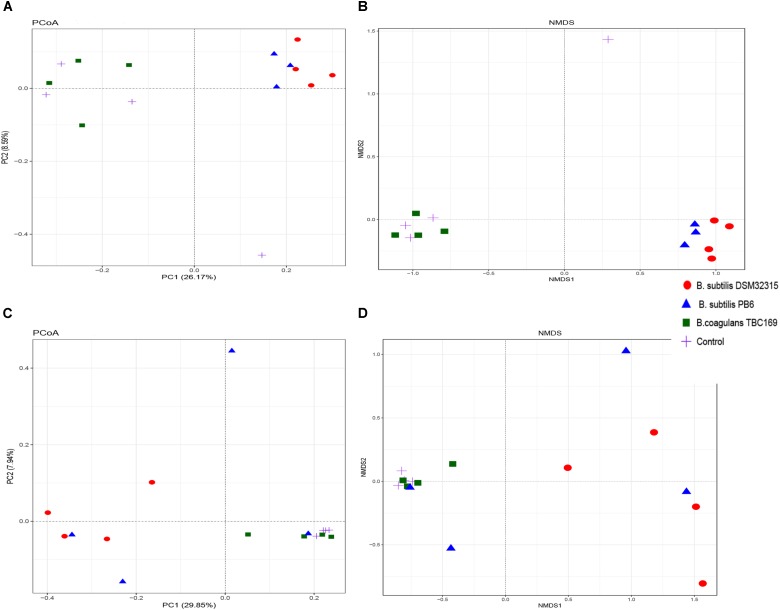
Structural comparison of jejunum microbiota among chicken with different probiotics supplementation. **(A)** Principal coordinate analysis plot of the jejunum microbiota based on the unweighted UniFracmetric on day 21. **(B)** Non-metric Multidimensional Scaling on day 21. **(C)** Principal coordinate analysis plot of the jejunum microbiota based on the unweighted UniFracmetric on day 42. **(D)** Non-metric Multidimensional Scaling on day 42.

The PCoA and NMDS plot of the jejunum microbiota were based on the weighted UniFrac metric measured as Adonis (*P* = 0.002 on day 21 and *P* = 0.012 on day 42) and Anosim (*P* = 0.003 on day 21 and *P* = 0.019 on day 42). These methods showed differences of chickens’ microbiota with different probiotics supplementation compared to that of the control (Figure 3). The compositions of the jejunal microbiota of the probiotics supplemented chicken were significantly different in comparison to that of the jejunal microbiota of the control.

### Impact of *Bacillus* spp. Supplementation on Microbial Diversity

Alpha diversity (sample OTU richness) including Simpson index and Shannon index (Table 6) was measured to detect the diversity and structure of jejunal microbial communities with different *Bacillus* spp. supplementations. According to Shannon and Simpson index, the number of *B. subtilis* PB6 and *B. subtilis* DSM32315 was higher (*P* < 0.032) than the control on day 21, which illustrated that the jejunum microbial diversity of broiler chickens was greater than the control. But, according to Shannon index, the number of *B. subtilis* PB6 and *B. subtilis* DSM32315 was higher (*P* < 0.05) than *B. coagulans* TBC169 group. By contrast, on day 42, the figure of the control was the most compared with other treatments, and that was higher (*P* < 0.05) than the supplementation with *B. subtilis* DSM32315.

**Table 6:** The effect of different sources of *Bacillus* spp. on diversity index of jejunum microbiota of broiler chickens on days 21 and 42^1^.

Items	Control	*Bacillus coagulans* TBC169	*Bacillus subtilis* PB6^2^	*Bacillus subtilis* DSM32315	*p*-value
**Day 21**					
Simpson	0.79 ± 0.03^a^	0.86 ± 0.01^abc^	0.93 ± 0.01^c^	0.91 ± 0.05^bc^	0.032
Shannon	3.87 ± 0.33^a^	4.40 ± 0.26^a^	6.14 ± 0.27^c^	6.64 ± 0.42^c^	0.000
**Day 42**					
Simpson	0.99 ± 0.00	0.89 ± 0.07	0.83 ± 0.16	0.54 ± 0.14	0.071
Shannon	9.13 ± 0.13^a^	7.12 ± 1.17^ab^	6.90 ± 1.65^ab^	3.23 ± 0.88^c^	0.018


*^a,b,c^Means within a row with no common superscripts differ significantly (P < 0.05). ^1^n = 4 replicates per treatment. ^2^n = 3 replicates per treatment on day 21.*

### Impact of *Bacillus* spp. on the Number of Indicated Taxonomic Rank

At different taxonomic levels (phylum, class, order, family, and genus), *B. subtilis* PB6 and *B. subtilis* DSM32315 increased (*P* < 0.01) the number of indicated taxonomic rank on day 21, whereas that decreased (*P* < 0.01) the number of indicated taxonomic rank on day 42. The number of indicated taxonomic rank of *B. coagulans* TBC169 group showed no significant differences (*P* > 0.05) compared with the control group on both days 21 and 42 (Table 7).

**Table 7:** The effect of different sources of *Bacillus* spp. on the number of indicated taxonomic rank of broiler chickens on days 21 and 42^1^.

Items	Control	*Bacillus coagulans* TBC169	*Bacillus.subtilis* PB6^2^	*Bacillus subtilis* DSM32315	*p*-value
**Day 21**					
Phylum	18.25 ± 2.56^ab^	15.75 ± 1.65^a^	25.33 ± 0.88^c^	25.50 ± 1.19^c^	0.005
Class	43.75 ± 12.09^ab^	34.00 ± 5.97^a^	71.67 ± 2.33^c^	75.50 ± 3.28^c^	0.005
Order	74.00 ± 19.83^a^	55.00 ± 8.22^a^	124.68 ± 1.86^cd^	132.75 ± 4.84^d^	0.002
Family	125.00 ± 27.39^a^	93.50 ± 10.04^a^	202.00 ± 3.61^c^	212.25 ± 9.36^c^	0.001
Genus	176.50 ± 33.44^a^	145.50 ± 11.51^a^	291.00 ± 4.04^c^	302.50 ± 14.33^c^	0.000
**Day 42**					
Phylum	40.00 ± 0.91^a^	36.00 ± 2.92^a^	28.00 ± 4.92^bc^	22.25 ± 2.87^c^	0.009
Class	108.75 ± 4.30^a^	97.25 ± 6.14^a^	78.50 ± 12.26^bc^	64.75 ± 8.23^c^	0.011
Order	155.00 ± 13.00^a^	150.50 ± 9.02^a^	114.50 ± 16.18^bc^	90.25 ± 11.85^c^	0.004
Family	190.25 ± 3.75^a^	181.25 ± 13.46^a^	137.25 ± 17.88^bc^	112.50 ± 13.37^c^	0.004
Genus	215.00 ± 3.03^a^	203.00 ± 19.06^a^	147.00 ± 24.02^bc^	111.25 ± 16.70^c^	0.004


*^a,b,c,d^Means within a row with no common superscripts differ significantly (P < 0.05). ^1^n = 4 replicates per treatment. ^2^n = 3 replicates per treatment on day 21.*

### Impact of *Bacillus* spp. Supplementation on the Gut Microbial Compositions

**FIGURE 4:**
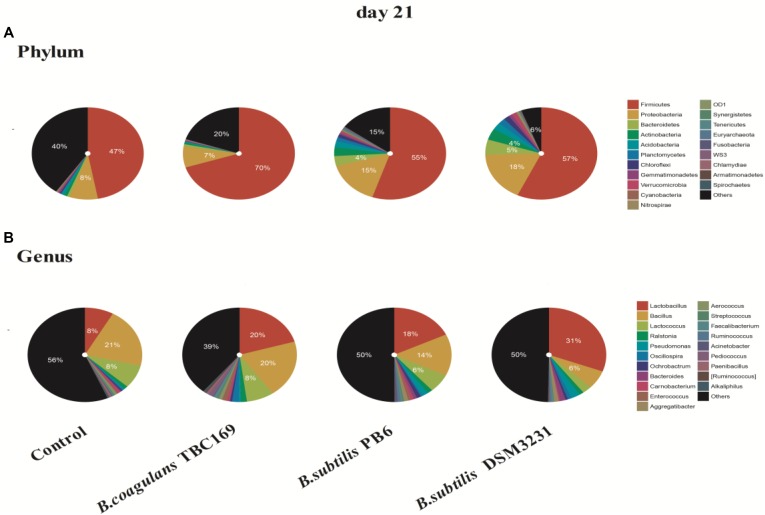
The effect of different sources of *Bacillus* spp. on gut microbiota composition at different levels (**A**: Phylum; **B**: Genus) on day 21.

As shown in Figure 4, the phylum level analysis demonstrated that three kinds of *Bacillus* spp. significantly (*P* < 0.05) influenced the percentage of Firmicutes, Proteobacteria, Bacteroidetes, Actinobacteria, and Acidobacteria on 21 days. The control and all treatments held the largest share in *Firmicutes* at around 50% (Figure 3). Meanwhile, the percentage of Firmicutes was higher (*P* < 0.01) in the *B. coagulans* TBC169 group (70.10%) than that in the control group (47.08%). As for the rest phyla (Proteobacteria, Bacteroidetes, and Actinobacteria), dietary supplementation with *B. subtilis* PB6 and *B. subtilis* DSM32315 group were higher (*P* < 0.05) than both the control and *B. coagulans* TBC169 group, and there were no differences (*P* > 0.05) between the control and *B. coagulans* TBC169 group. *Lactobacillus*, which belongs to the phylum of Firmicutes, was the most abundant genus in chicken jejunal microbiota with three kinds of *Bacillus* spp. supplementation, followed by *Bacillus, Lactococcus*, and *Bacillaceae* on day 21. Interestingly, Three kinds of *Bacillus* spp. tended to decrease (*P* = 0.077) the percentage of *Bacillus* (phylum of Firmicutes), which was the most abundant genus in the microbiota of chicken jejunum in the control group at the same situation. Dietary supplementation with *B. subtilis* DSM32315 improved (*P* < 0.01) the composition of *Clostridiales* and *Pseudomonas* compared control group and *B. coagulans* TBC169 group at genus level.

**FIGURE 5:**
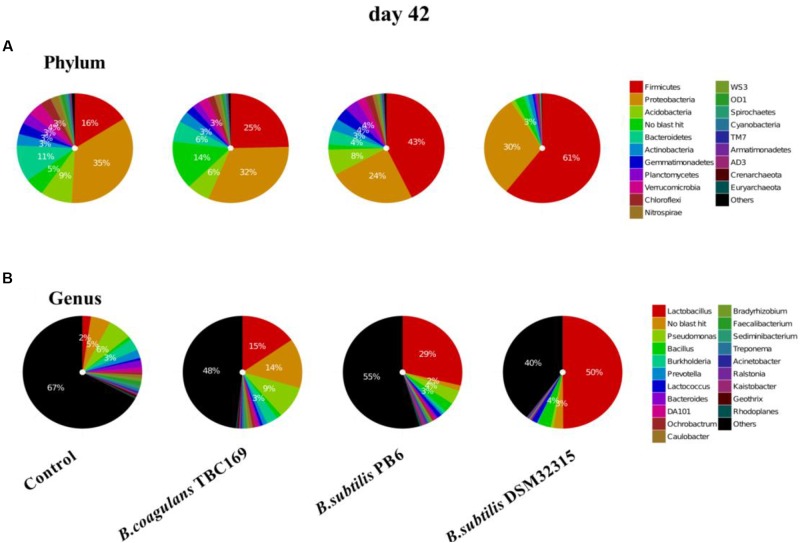
The effect of different sources of *Bacillus* spp. on gut microbiota composition at different levels (**A**: Phylum; **B**: Genus) on day 42.

The composition of jejunal microflora obviously changed with time. On day 42, the percentage of the phylum of Firmicutes dropped compared with on day 21, but it still occupied the dominant position in *B. subtilis* PB6 and *B. subtilis* DSM32315 groups, followed by Proteobacteria and Acidobacteria (Figure 5). But, the percentage of phylum of Proteobacteria occupied the dominant position in control group and *B. coagulans* TBC169 group, followed by Firmicutes and Acidobacteria. Similarly, *Lactobacillus* was also the most abundant genus in jejunal microbiota of chicken with three kinds of *Bacillus* spp. supplementation on day 42. On the ability of decreasing the percentage of *Pseudomonas* and *Burkholderia, B. subtilis* PB6 and *B. subtilis* DSM32315 were stronger than *B.subtilis* TBC169, whereas *B.subtilis* DSM32315 was stronger than *B. subtilis* TBC169 and *B. subtilis* PB6 in decreasing the percentage of *DA101*. The supplementation with probiotics *B. subtilis* DSM32315 decreased (*P* < 0.05) the percentage of *Pseudomonas*, *Burkholderia*, *Prevotella*, *DA101* than control group, whereas the supplementation with probiotics *B. subtilis* PB6 decreased (*P* < 0.05) the percentage of *Burkholderia*, *Prevotella*, *DA101* than control group.

**FIGURE 6:**
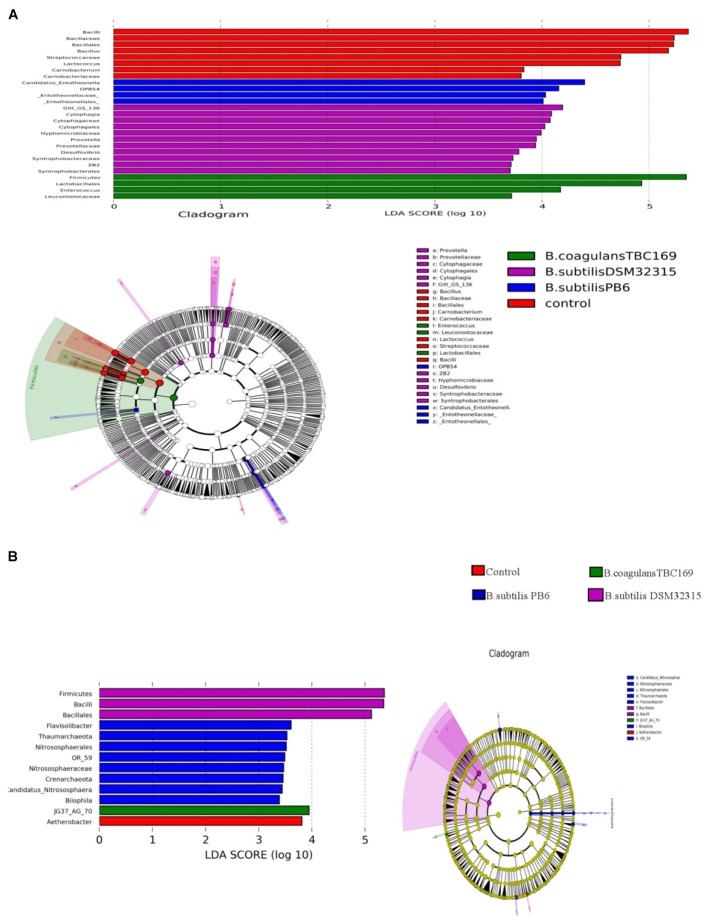
Significantly different taxa between different probiotic stains and control on day 21 **(A)** and day 42 **(B)**.

**FIGURE 7:**
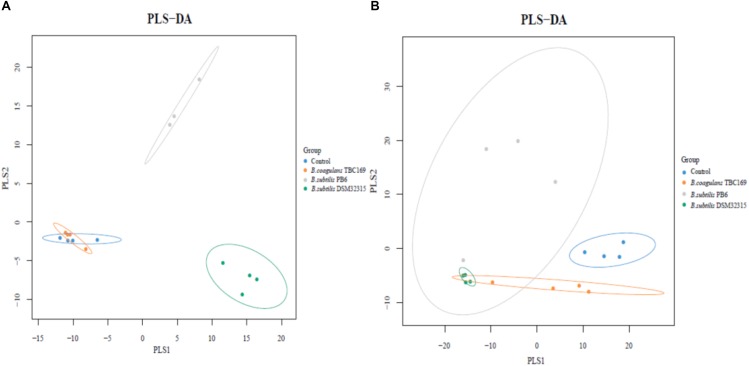
PLS-DA analysis of jejunal digesta collected on day 21 **(A)** and day 42 **(B)**.

A cladogram representative of the structure of the jejunum microbiota and the predominant bacteria were shown in Figure 6. LEfSe detected a marked increase (LDA score > 2) in the relative abundance among the four treatment groups. PLS-DA analysis showed some significant differences in the bacterial composition at genus levels among four treatment groups (Figure 7). It shows that bacterial composition on day 21 (Figure 7A) and 42 (Figure 7B) exhibiting the tendency of separation in the profiles among the four treatments and indicating the degree of reliability of PCoA analysis.

**FIGURE 8:**
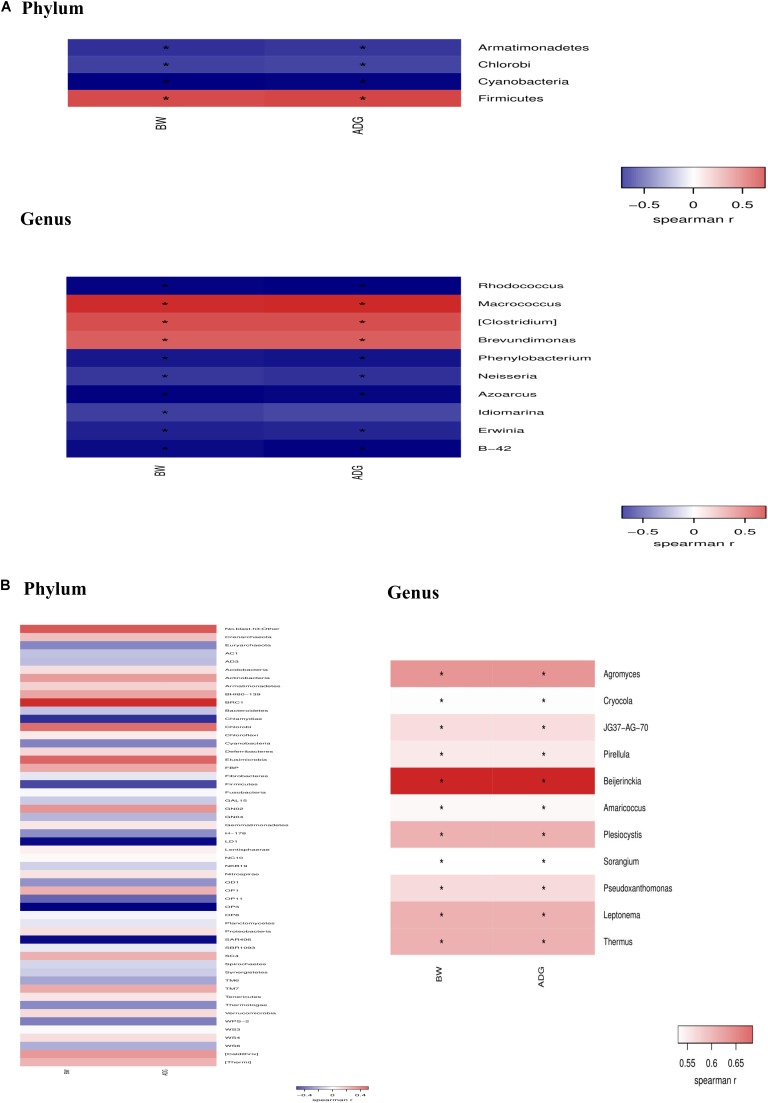
Spearman’s rank correlation coefficient between taxonomic change of jejunal microflora because of dietary *Bacillus* spp. supplementation and growth performance (BW and ADG) on day 21 **(A)** and day 42 **(B)**.

Data (Figure 8) from spearman correlation coefficients showed that change of Firmicutes have significantly (*P* < 0.05) positive correlations with growth performance (i.e., BW and ADG), whereas Armatimonadets, Chlorobi, and Cyanobacteria had significantly (*P* < 0.05) negative correlations with BW and ADG at the phylum level on day 21. The changes of *Macrococcus*, *Clostridium* and *Brevundimonas* have positive (*P* < 0.05) correlations with BW and ADG, whereas *Rhodococcus*, *Phenylobacterium*, *Neisseria*, *Azoarcus*, *Idiomarina*, *Erwinia*, *B-42* have significantly negative (*P* < 0.05) correlations with BW and ADG at the genus level on day 21. *Agromyces, Cryocola, JG37-AG-70, Pirellula, Beijerinckia, Amaricoccus, Plesiocystis, Sorangium, Pseudoxanthomonas, Leptonema*, and *Thermus* have positive (*P* < 0.05) correlations with BW and ADG at the genus level on day 42.

## Discussion

Due to the high biological safety and beneficial functions in the regulation of intestinal microflora and micro-ecological balance, *B. subtilis* and *B. coagulans* have been widely used in the field of animal feed (Selvam et al., 2009; Hung et al., 2012; Pandey and Vakil, 2016). Previous studies indicated that the addition of *B. subtilis* and *B. coagulans* as probiotics to broilers’ diets can obviously improve the growth performance and FCR (Hung et al., 2012; Jeong and Kim, 2014; Lee K.-W. et al., 2014; Hossain et al., 2015; Li et al., 2016). In this study, *B. subtilis* PB6, *B. subtilis* DSM32315 and *B. coagulans* TBC169 were used for probiotic supplementations in chickens’ diets. Although there was no significant difference in ADFI between birds fed diet with probiotics and the control group at the age of 3rd and 6th week, the ADG and BW of birds fed with *B. coagulans* TBC169 diets were higher than that of chickens fed with the basal diets (Table 2) (*P* < 0.05). Generally, the reason why the addition of *Bacillus* spp. can improve the growth performance of broilers is that these probiotics may regulate the composition of intestinal microflora, and exert antibiotics and organic acids to inhibit the survival and colonization of harmful bacteria and further facilitate the balance of intestinal micro-ecosystem (Pedroso et al., 2006). However, the supplementation with *B. subtilis* did not affect the growth performance of chickens including the indexes of ADFI, ADG, and FCR (Table 2) (*P* > 0.05). In addition, there were also reports that dietary *B. subtilis* failed to improve ADG and decrease FCR in broiler chickens (Knap et al., 2011; Lee K.-W. et al., 2014). The reasons such as the bacterial strains, animal conditions, methods of using probiotics and environmental factors may also contribute to these results (Landy and Kavyani, 2013; Lee S.H. et al., 2014).

In addition, another characteristic of *Bacillus* spp. is that they can produce various digestive enzymes such as proteases, celluloses and amylases etc. and promoting the transformation of pepsinogen into protease after colonizing in intestinal tract of hosts. They can further stimulate the peristalsis of the intestine to improve nutritional digestion (Giang et al., 2011), which is related to the improvement of broilers’ growth performance. A xylanase gene from the chicken caecum has been isolated and over expressed which focused on the potential development of novel and optimized feed additives for industrial use (Al-Darkazali et al., 2017). *B. coagulans* can produce bacteriocins, which have been widely reported to exhibit antibacterial function in various models (Riazi et al., 2009; Nobutani et al., 2017). Meanwhile, *B. coagulans* is associated with lactic acid and other organic acids production, while lactic acid can inhibit gut colonizations of harmful bacteria (Cui et al., 2005) and activate the peristalsis in the intestine to mediate nutrient digestion (Giang et al., 2010; Ma et al., 2014). More recently, *B. coagulans*-produced dysprosium has been identified to exhibit broad antibacterial spectrum (Honda et al., 2011). Also, *B. coagulans* can maintain gut microbiota balance via breaking down polysaccharides into oligosaccharides (Zheng et al., 2011). The apparent digestibility of CP and GE were higher for birds in the *B. coagulans* TBC169 treatment group compared with those in the control (*P* < 0.05) in the present study. The ability of improving the apparent total tract digestibility of CP, *B.subtilis* TBC169 was even stronger than *B. subtilis* PB6. Dietary *B. subtilis* DSM32315 supplementation influenced the apparent digestibility of GE (Table 3) (*P* = 0.05). This was in accordance with the findings of Pelícia et al. (2004) who suggested that the improvement in broiler growth performance was likely linked to a better ileal digestibility of nutrients. Similar research reported the increasing levels of dietary supplementation with *B. subtilis* LS 1–2 product was linked to the improvement in the retention of DM, GE, and CP in broilers (Sen et al., 2012). The dietary supplementation with *Bacillus* spp. can improve GE and CP digestibility, which is highly associated with gut health and subsequent digestive capacity. The increased VH and VH/CD ratio were directly correlated with an enhanced epithelial turnover (Sen et al., 2012) and high VH and VH/CD ratio suggested an improved intestinal nutrient digestibility and absorption capacity (Montagne et al., 2003). The present study showed that *B. coagulans* TBC169 increased (*P* < 0.07) VH or VH/CD ratio mainly in jejunum compared with the control diet at different phases (Table 4). The *B. coagulans* TBC169 supplementation might improve the gut structure and further resulted in a greater absorption surface and high-speed turnover of epithelial cells. Similarly, an increase of VH/CD ratio was observed in broilers fed with *B.* *coagulans* (Hung et al., 2012). This situation was also consistent with the FCR and BW results in the *B. coagulans* TBC169 group, which indicated the positive growth performance might be associated with good intestinal morphology. However, at the same condition, the dietary *B. subtilis* PB6 supplementation improved VH and VH/CD ratio only in jejunum compared with the control diet on day 42. The dietary *B. subtilis* DSM32315 supplementation did not affect the structure of intestine compared with the control on days 21 and 42 (Table 4). Therefore, different *Bacillus* spp. has distinguished effects on stimulating the differentiation and proliferation of intestinal epithelial cells and improving nutrient utilization.

This study found that *B. subtilis* PB6 and *B. subtilis* DSM32315 more strongly affected the number of indicated taxonomic rank in jejunum microflora than the control or *B. coagulans* TBC169 group (Table 7). Furthermore, it is generally agreed that the improvement in growth performance and feed conversion efficiency rely on a healthy intestinal morphology, which may be related to a balance of entire intestinal microflora, resulting in a better intestinal environment (Pedroso et al., 2006; Mountzouris et al., 2010). High-throughput sequencing of the V4 region of the 16S rRNA gene was used for detecting the jejunum microbiota of individual broiler chickens fed diets without or with *Bacillus* spp. supplementation in the current study. *B. subtilis* PB6 and *B. subtilis* DSM32315 treatments exhibited more OTUs than the control on day 21, but the OUTs were higher (*P* < 0.005) in the control compared with other treatments on day 42 (Table 5). In addition, the number of indicated taxonomic rank varied in accordance with OUT variation (Table 7). As the researches exploring the relationship between the growth performance and intestinal microbial composition have become more popular, recent reports showed that alpha index was strongly correlated with diversity (Martiny et al., 2011). Many studies suggested that gut microbiota with high diversity could be more stable and healthier than those with low diversity (Konstantinov et al., 2004). Simpson and Shannon indexes can reflect the community diversity of the microbiota. In this study, Simpson and Shannon indexes showed the similar trend to the number of OTUs (Table 6). *B. subtilis* PB6 and *B. subtilis* DSM32315 treatments enhanced the diversity of jejunal microbiota on day 21 compared with the control (*P* ≤ 0.032). Diet probiotic supplementation influenced diversity of the microbiota according to Simpson and Shannon indexes (*P* = 0.071 and *P* = 0.018) on day 42. Simpson and Shannon index are respond to *Bacillus* spp. were different on days 21 and 42. The number of Shannon in the treatments with *B. subtilis* DSM32315 was lower (*P* < 0.05) than that in the control. Nevertheless, *B. coagulans* TBC169 strains did not influence the diversity of jejunal microbiota on days 21 and 42. Changes in taxonomic diversity are the most used indicator to infer changes in microbiological activity, but it is becoming apparent that many of the functions of a normal microbiome can be carried out by a number of microbial groups (Kurokawa et al., 2007; Human Microbiome Project Consortium, 2012). Many studies have shown that the microbial diversity of the chicken microbiota is relatively lower compared to the intestinal microbiota of other animals, which is attributed to the rapid transit of food through the digestive system, with short retention times (Wei et al., 2013). Microbial diversities increased during chicken development, reaching at the peak approximately on day 14 for the foregut and then remaining stable or decreasing slightly thereafter (Huang et al., 2018). Data from a previous study suggest a microbiome more affected by age rather than treatment (Ballou et al., 2016).

The complex microbiota colonized in a chicken’ GIT with more than over 900 different bacterial species (Torok et al., 2011). Basically, the most abundant phylum of the chicken intestinal microbiota is Firmicutes (44–55%), followed by Proteobacteria and Bacteroidetes, which is consistent with the present study (Figures 3, 4) (Qu et al., 2008). However, different sections GIT of chickens are highly interconnected and thus also influence each other’s microbiota compositions (Sklan et al., 1978). The composition and function of these communities has been shown to vary depending on the age of the birds, location in the GI tract and on the dietary components (Oakley et al., 2014; Pan and Yu, 2014; Oakley and Kogut, 2016. In addition, the variability in results may be due to sample types (feces vs. cecum), and/or conventional microbiological and molecular methods that have limited coverage and accuracy. The jejunum of a chicken was dominated by *Lactobacillus* species, mainly *L. salivarius* and *L. aviarius* (Gong et al., 2007; Zotta et al., 2017). According to a previous report, at the genus level, *Lactobacillus* is the predominant genus in the foregut, *Lactobacillus* provides nutrients to the host and defends against opportunistic pathogens (Huang et al., 2018). Nutrient absorption mainly occurs in the intestine where occupies by a large number of *Lactobacillus* spp. (Witzig et al., 2015). Various trials have demonstrated that probiotics can positively modulate the composition of the gut microflora of chickens via the stimulation of potentially beneficial populations and the reduction of potentially pathogenic bacteria (Higgins et al., 2008; Hussain et al., 2017b). Dietary inclusion of *B. subtilis* PB6 and *B. subtilis* DSM32315 mainly decreased the number of *Bacillus* genus bacterial and increased *lactobacilli* population both on days 21 and 42 compared with the control (Figures 3, 4). This phenomenon likely illustrated that the three probiotic treatments can regulate the intestinal bacterial flora by increasing the quantity of beneficial bacterial such as lactobacilli and decreasing the number of harmful bacterial like coli bacillus etc. Study have also shown that *Bacillus* spp. regulated the composition of intestinal bacterial flora, maintained the balance of GIT microflora and improved the immune function of the intestinal mucosa (Isolauri et al., 2001; Barbosa et al., 2005).

Animals with high FCR exhibited a higher abundance of *Acinetobacter*, *Bacteroides*, *Streptococcus*, *Clostridium* and *Lactobacillus* whereas *Escherichia*, *Shigella*, and *Salmonella* were more abundant in low FCR animals (Singh et al., 2014). Although the supplementation with probiotics *B. subtilis* DSM32315 increased (*P* < 0.05) the percentage of Clostridiales than control group on day 21, which did not affect the FCR in the present study. Individual FCR in broiler growers appears to vary, which may in part be due to variation in their gut microbiota. In this paper we analyzed the jejunal microbiota whereas most of the other studies analyzed fecal and cecal microorganisms. *Clostridium* spp., *Enterococcus* spp., *Streptococcus* spp., and *Bacteroides* spp. were shown to have polysaccharide degrading activity against non-starch polysaccharide (NSPs) found in grain (Beckmann et al., 2006). In mice and humans, Firmicutes have been shown to have a positive relationship with the ability to harvest energy from the diet (Turnbaugh et al., 2006; Jumpertz et al., 2011), and the Firmicutes/Bacteroides ratio may also be important for optimum physiology and nutrition (Bervoets et al., 2013). An increase in fecal Firmicutes was associated with an increase in nutrient absorption, whereas an increase in fecal Bacteroidetes was associated with a decrease in nutrient absorption (Jumpertz et al., 2011). Similar experiments on pig and poultry showed that the increase of Firmicutes abundance and the decrease of Bacteroidetes abundance were positively correlated with the increase of host weight and fat deposition, indicating that intestinal microorganisms were related to the absorption and utilization of energy by the host (Angelakis and Raoult, 2010). In the lowest portion of the small intestine, *Lactobacillus* spp. have been implicated as a causal factor in low performance (DeLange and Wijtten, 2010), suggesting the location of colonization by probiotic strains may affect performance.

There were many consistencies among the indexes determined in this study. Dietary supplementation with three probiotics mostly showed the beneficial effects on chicken’s growth and intestinal health. Among that, the *B. coagulans* TBC169 group showed the better growth performance, nutrients digestibility and intestinal morphology compared with the two *B. subtilis* treatments, while two *B. subtilis* treatments presented more positive variation of the jejunum microflora of chickens than that in the *B. coagulans* TBC169 group (Figure 7). The possible explanations might be the different characteristics of different strains, the environmental and individual differences etc. In addition, the investigation of jejunum microflora of chickens might not stand for the whole gut microbiota conditions. Therefore, the internal relationships and underlying mechanism are worth further exploring.

## Conclusion

Dietary addition of three probiotic *Bacillus* spp. strains affect body weight and intestinal morphology through altering intestinal microbiota composition in broiler chickens. The findings highlight the importance of intestinal microbiota in mediating the various physiological functions of probiotics in the host. However, the effect of different strains of *Bacillus* on intestinal microbial composition was different.

## Author Contributions

All authors listed have made a substantial, direct and intellectual contribution to the work, and approved it for publication.

## Conflict of Interest Statement

The authors declare that the research was conducted in the absence of any commercial or financial relationships that could be construed as a potential conflict of interest.
