# Corrigendum: Intestinal Morphologic and Microbiota Responses to Dietary *Bacillus* spp. in a Broiler Chicken Model

**DOI:** 10.3389/fphys.2019.00332

**Published:** 2019-04-02

**Authors:** Cheng-liang Li, Jing Wang, Hai-jun Zhang, Shu-geng Wu, Qian-ru Hui, Cheng-bo Yang, Re-jun Fang, Guang-hai Qi

**Affiliations:** ^1^College of Animal Science and Technology, Hunan Agricultural University, Changsha, China; ^2^Key Laboratory of Feed Biotechnology of Ministry of Agriculture and Rural Affairs, Feed Research Institute, Chinese Academy of Agricultural Sciences, Beijing, China; ^3^Department of Animal Science, Faculty of Agricultural and Food Sciences, University of Manitoba, Winnipeg, MB, Canada

**Keywords:** probiotics, growth performance, intestinal morphology, jejunum microbiota, broiler

In the original article, there was a mistake in Figure 1 as published. Due to poor image quality a new image was prepared during the production stage. Unfortunately, the incorrect image was uploaded and used in the published article. The corrected Figure 1 appears below.

**Figure 1:**
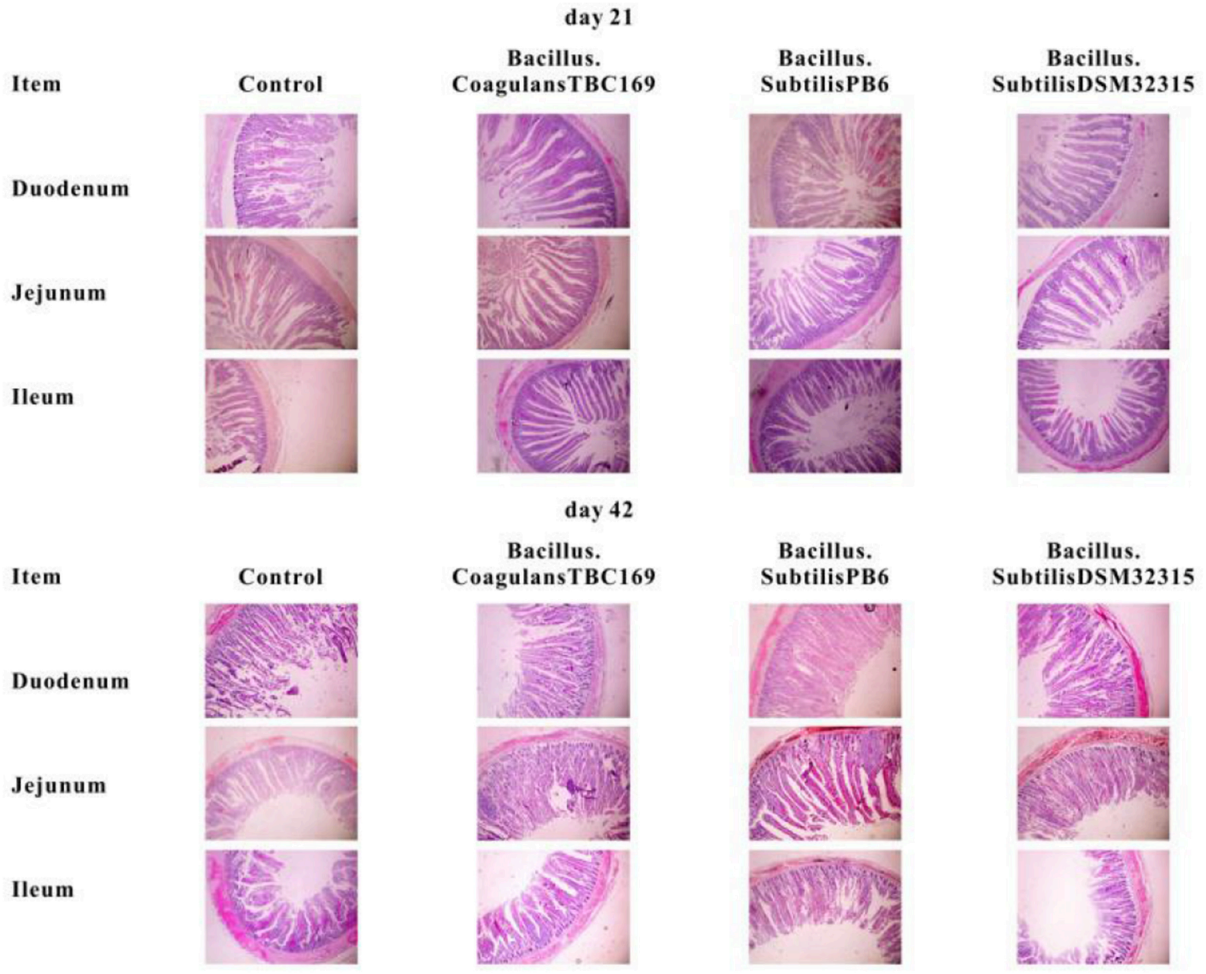
Slice of jejunum morphology (40 ×) of broiler chickens on day 21 and day 42.

Additionally, there was a mistake in Figure 6 as published. We have uploaded Figure 6A was uploaded during production of the article instead of Figure 6. Thus, Figure 6 should include both Figures 6A,B. The corrected Figure 6 appears below.

**Figure 6:**
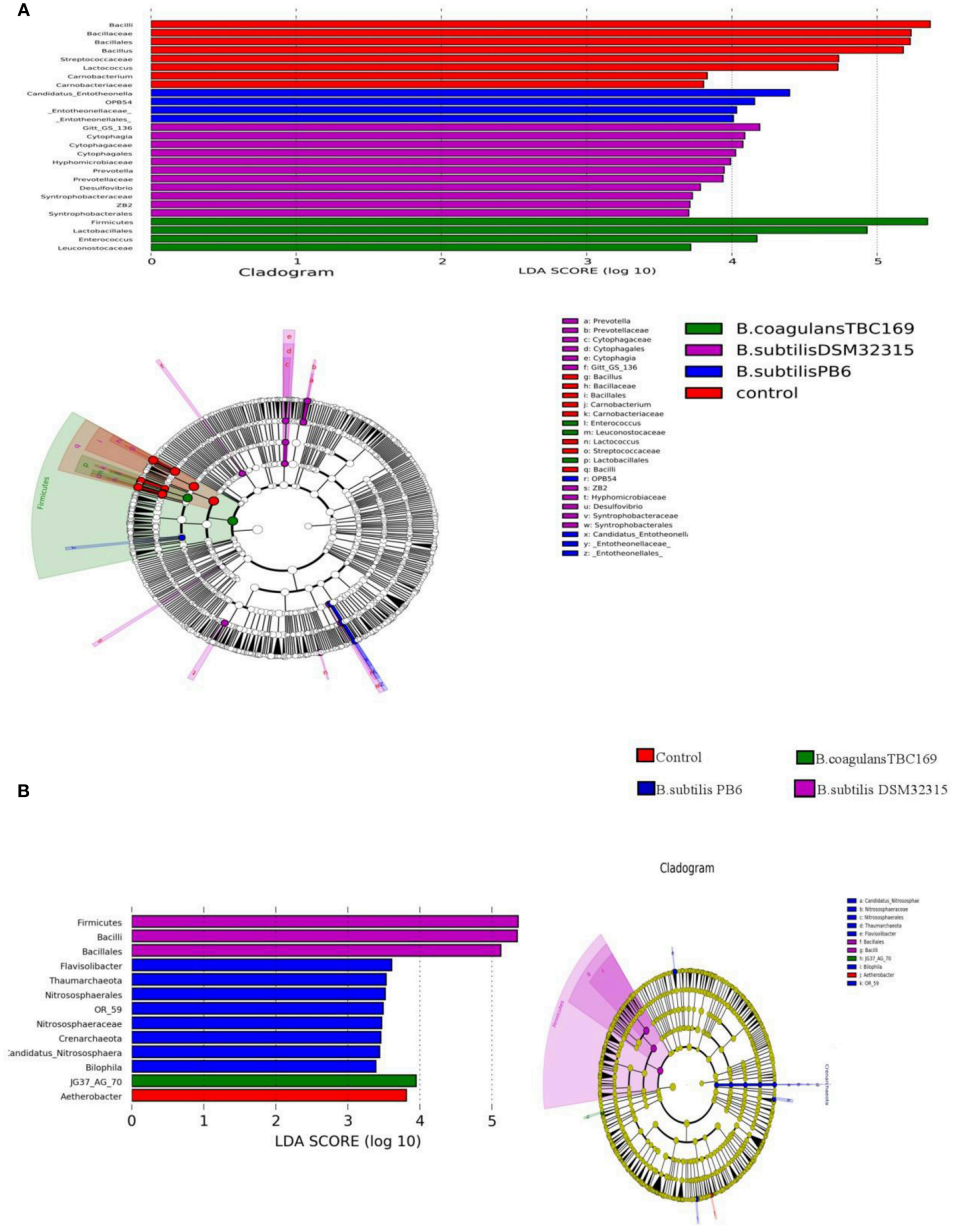
Significantly different taxa between different probiotic stains and control on day 21 **(A)** and day 42 **(B)**.

The authors apologize for this error and state that they do not change the scientific conclusions of the article in any way. The original article has been updated.

